# Tyrosine 402 Phosphorylation of Pyk2 Is Involved in Ionomycin-Induced Neurotransmitter Release

**DOI:** 10.1371/journal.pone.0094574

**Published:** 2014-04-09

**Authors:** Zhao Zhang, Yun Zhang, Zheng Mou, Shifeng Chu, Xiaoyu Chen, Wenbin He, Xiaofeng Guo, Yuhe Yuan, Masami Takahashi, Naihong Chen

**Affiliations:** 1 State Key Laboratory of Bioactive Substances and Functions of Natural Medicines, Beijing Key Laboratory of New Drug Mechanisms and Pharmacological Evaluation Study, Department of Pharmacology, Institute of Materia Medica, and neuroscience center, Chinese Academy of Medical Sciences & Peking Union Medical College, Beijing, People’s Republic of China; 2 Basic Medical College, Shanxi University of Traditional Chinese Medicine, Taiyuan, People’s Republic of China; 3 Department of Biochemistry, Kitasato University School of Medicine, Sagamihara, Kanagawa, Japan; Vanderbilt University Medical Center, United States of America

## Abstract

Protein tyrosine kinases, which are highly expressed in the central nervous system, are implicated in many neural processes. However, the relationship between protein tyrosine kinases and neurotransmitter release remains unknown. In this study, we found that ionomycin, a Ca^2+^ ionophore, concurrently induced asynchronous neurotransmitter release and phosphorylation of a non-receptor protein tyrosine kinase, proline-rich tyrosine kinase 2 (Pyk2), in clonal rat pheochromocytoma PC12 cells and cerebellar granule cells, whereas introduction of Pyk2 siRNA dramatically suppressed ionomycin-induced neurotransmitter release. Further study indicated that Tyr-402 (Y402) in Pyk2, instead of other tyrosine sites, underwent rapid phosphorylation after ionomycin induction in 1 min to 2 min. We demonstrated that the mutant of Pyk2 Y402 could abolish ionomycin-induced dopamine (DA) release by transfecting cells with recombinant Pyk2 and its mutants (Y402F, Y579F, Y580F, and Y881F). In addition, Src inhibition could prolong phosphorylation of Pyk2 Y402 and increase DA release. These findings suggested that Pyk2 was involved in ionomycin-induced neurotransmitter release through phosphorylation of Y402.

## Introduction

Neuronal communication in the mammalian brain mainly depends on the release of neurotransmitters [1]–[3], which are stored in synaptic vesicles? Neurotransmitters are secreted across the narrow space between pre-synaptic and post-synaptic membranes in response to an increase in calcium concentrations after effective stimulation occurs. The regulation of neurotransmitter release is postulated to be the basis of human higher nervous activity and one of the important mechanisms of synaptic plasticity underlying learning and memory [4]. Neurotransmitter disorders generally cause fulminant neurodegeneration in neurons [5].

Studies have shown that various protein tyrosine kinases (PTKs) are expressed in the mammalian central nervous system (CNS) [6]–[8]. Many PTKs exhibit key functions in neuronal development and synaptic plasticity. Pyk2, a non-receptor PTK [9], also known as focal adhesion kinase 2 (FAK-2) [10], and calcium-dependent tyrosine kinase (CADTK) [11] (for convenience, hereafter referred to as Pyk2), is a second member of the FAK subfamily and was first reported by Avraham in 1995 [9], [12], [13]. Pyk2 is preferentially expressed in neuronal and hematopoietic cells. Pyk2 and FAK, which is also a member of the FAK family, share approximately 48% amino acid identity and 65% similarity [9], [12]–[16], and have similar domain structures that include an N-terminal FERM (Protein4.1-Ezrin-Radixin-Moesin) domain, a centrally located kinase domain, two proline-rich regions in the C-terminus, and a focal adhesion targeting domain [16]. The tyrosine 402 on the linker between the FERM domain and catalytic domain is the main autophosphorylation site of Pyk2 [9], which serves as a docking site for src family kinase (SFK) upon phosphorylation. Recruited SFK can phosphorylate additional tyrosine residues within Pyk2 (Tyr-579, 580, and 881), which results in enhanced catalytic activity and provides docking sites for SH2 domain-containing proteins [16]–[18].

Pyk2 autophosphorylation has a key function in multiple intracellular signals, including the activation of migration in macrophages [19] and cancer cells [20], [21], and bone resorption in osteoclasts [22], [23]. Pyk2 contributes to neurite outgrowth and synaptic plasticity [24]–[28] in the CNS. It has been demonstrated that Pyk2 acts as a signaling link for induction of long-term potentiation (LTP) in CA1 hippocampus. Pyk2 activation is required for downstream activation of Src, which upregulates the activity of NMDA receptors in this process [24]. However, intracellular application of K457A Pyk2, a catalytically inactive mutant [9], can produce a short-lasting post-tetanic potentiation (PTP) upon tetanic stimulation [24], though LTP fails to be induced. Activated Pyk2 has been detected in cerebral ischemia and epilepsy. Pyk2 undergoes rapidly phosphorylation in cortical neurons after cerebral ischemia in response to increased Ca^2+^ influx, thereby contributing to neuronal cell apoptosis and death [29].

The increase in intracellular calcium is believed to be a major cause of neurotransmitter release and activation of Pyk2. Thus, Pyk2 may be involved in neurotransmitter release in response to increased Ca^2+^ influx. At low concentrations, ionomycin, a Ca^2+^ ionophore, can increase intracellular Ca^2+^ concentrations in neurites at a higher extent than in neuronal cell bodies, resulting in early neurite degeneration [30]. In this study, ionomycin was used to imitate intracellular Ca^2+^ overload. Results showed that ionomycin-induced neurotransmitter release was dependent on Y402 phosphorylation of Pyk2. Src, as a downstream signal molecule of Pyk2, exhibits an antagonistic action during neurotransmitter release. Src inhibition may prolong the duration of neurotransmitter release by extending the phosphorylation time of Pyk2 on Y402. These findings suggested that Pyk2 was involved in neurotransmitter release through Y402 phosphorylation.

## Results

### Changes in Pyk2 Phosphorylation are Concurrent with Neurotransmitter Release

**Figure 1:**
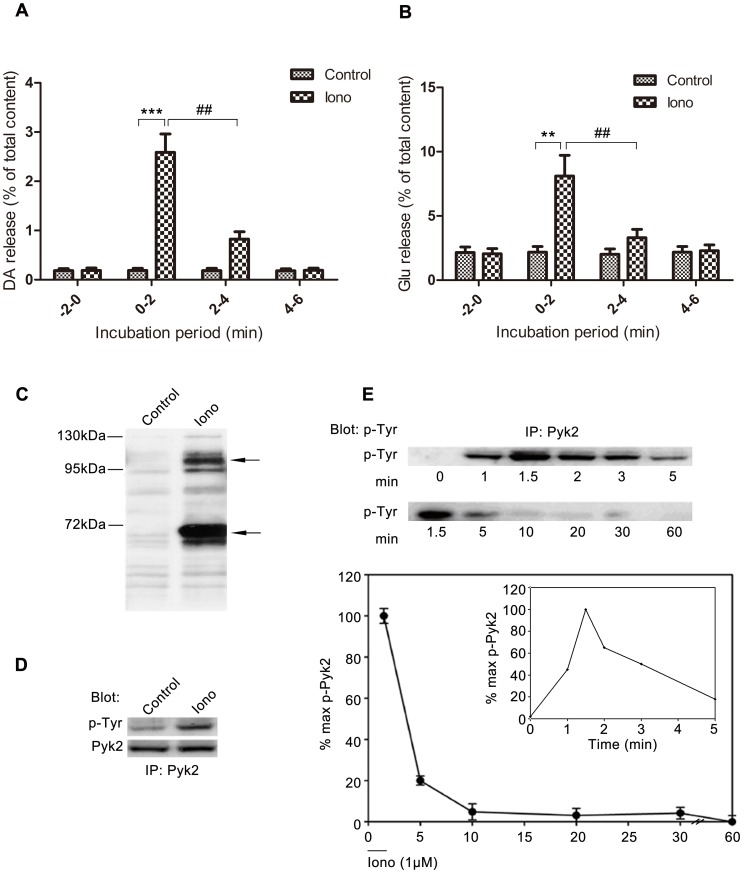
Pyk2 phosphorylation is consistent with ionomycin-induced neurotransmitter release. (**A**) PC12 cells were incubated for 2 min in low-K^+^ solution. DA release during this period represented the basal release. The buffer was immediately changed every 2 min with the low-K^+^ solution with or without 1 μM ionomycin to stimulate Ca^2+^ influx. Samples were collected and tested according to the release assay in Materials and Methods. The amount of DA release in the medium was expressed as the percentage of total cellular content (n = 8/sample). (**B**) Cerebellar granule cells were performed similar to PC12 cells, and the amount of Glu released in the medium was expressed as the percentage of total cellular content (n = 8/sample). (**C**) PC12 cells were incubated with or without 1 μM ionomycin, lysed, and subjected to western blot using an anti-phosphotyrosine (P-Tyr, 4G10) antibody. Arrows indicate the major immunoreactive bands co-migrating with Pyk2 (upper) and paxillin (lower). (**D**) PC12 cell lysates were subjected to IP with anti-Pyk2 antibody, followed by IB with anti-phosphotyrosine and anti-Pyk2 antibodies. (**E**) PC12 cells were incubated for 2 min at 37°C in low-K^+^ solution with or without 1 μM ionomycin. Equal amounts of cell lysates were immunoprecipitated with anti-Pyk2 antibody, and immunoblotted with phosphotyrosine antibody. Phosphorylation levels were studied in different experiments from 0 min to 5 min (right, inset) or from 1.5 min to 60 min (left, main graph). Pyk2 phosphorylation at each indicated time is expressed as a percentage of the maximal level of induced phosphorylation. Note: Duration of ionomycin treatment is indicated by the horizontal bar, which represents 2 min. The values in the results are expressed as mean ± S.E.M. from four representative experiments (^##^p<0.01, **p<0.01, ***p<0.001).

Ca^2+^ triggers neurotransmitter release in at least two principal modes, synchronous and asynchronous release [31]–[33]. Ionomycin triggers asynchronous release by increasing bulk “residual” Ca^2+^ in cells. Fig. 1A shows that 1 μΜ ionomycin induced significant DA release from PC12 cells (***p<0.001 iono *vs*. control at 0 min to 2 min, Student’s *t* test). DA release reached its peak in 0 min to 2 min, and declined immediately in 2 min to 4 min (iono treated groups, one way ANOVA, *post-hoc* Newman-Keuls, F_3,28_ = 229.8, ^##^ *p*<0.01 ionomycin stimulated at 0 min to 2 min *vs*. ionomycin stimulated at 2 min to 4 min). Ionomycin-induced Glu release from cerebellar granule cells (CGNs) showed similar changes (Fig. 1B) with DA release from PC12 cells (iono treated groups, one way ANOVA, *post-hoc* Newman-Keuls, F_3,28_ = 32.73, ^##^ *p*<0.01 ionomycin stimulated at 0 min to 2 min *vs*. ionomycin stimulated at 2 min to 4 min).

To identify the intracellular tyrosine-phosphorylated proteins involved in ionomycin-induced neurotransmitter release, PC12 cells incubated with 1 μΜ ionomycin were collected and tested by western blot using an anti-phosphotyrosine antibody. Fig. 1C shows that treatment with 1 μΜ ionomycin for 2 min induced several tyrosine-phosphorylated proteins in the cellular homogenate of PC12 cells, especially at 55 kDa to 70 kDa and 110 kDa to 130 kDa. The molecular masses of two major phosphotyrosine immunoreactive bands were similar to those of Pyk2 (120 kDa) and paxillin (68 kDa). Pyk2 is abundant in the CNS [24]–[28], and is implicated in diverse cellular events. Thus, Pyk2 was selected as a candidate in this study. Ionomycin-induced tyrosine phosphorylation of Pyk2 was demonstrated by immunoprecipitation (IP) with anti-Pyk2 antibody followed by immunoblotting (IB) with anti-phosphotyrosine antibody (Fig. 1D). Fig. 1E demonstrates that ionomycin-stimulated Pyk2 phosphorylation in PC12 cells peaked rapidly at 1.5 min, decreased to less than half maximum by 5 min, and quickly returned to the baseline level, which suggests that ionomycin-induced Pyk2 phosphorylation occurred in a transient and time-dependent manner. Thus, changes in Pyk2 phosphorylation occurred concurrently with neurotransmitter release.

### Pyk2 siRNA Abolishes Ionomycin-induced Neurotransmitter Release

**Figure 2:**
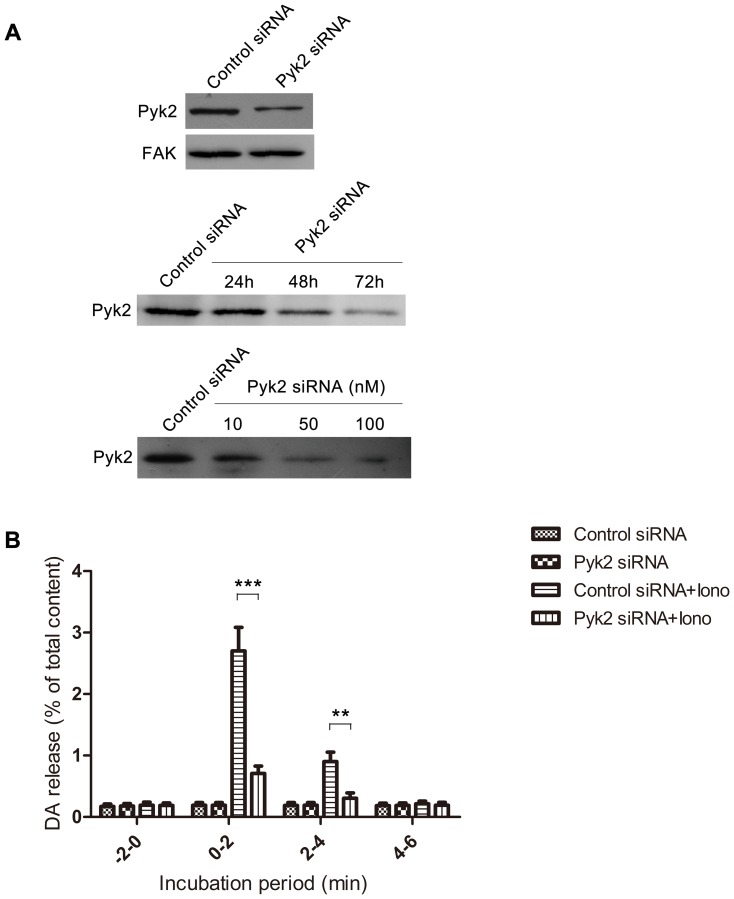
Pyk2 siRNA abolishes ionomycin-induced neurotransmitter release. (**A**) PC12 cells were transfected with 50 nM Pyk2 siRNA or control siRNA for 48 h, and tested for the expression of Pyk2 and FAK. PC12 cells were transfected with 50 nM Pyk2 siRNA for 24, 48, and 72 h or 50 nM control siRNA for 72 h, and harvested for western blot. PC12 cells were transfected with 10, 50, and 100 nM Pyk2 siRNA or 100 nM control siRNA for 48 h, and harvested for western blot. (**B**) PC12 cells were transfected with 100 nM Pyk2 siRNA or control siRNA for 48 h, and used for release assay with or without 1 μM ionomycin for 2 min. Samples were collected and tested for DA release. The amount of DA released in the medium was expressed as the percentage of total cellular content (n = 8/sample). The values are expressed as mean ± S.E.M. from four representative experiments (**p<0.01, ***p<0.001).

Pyk2 expression was inhibited by the introduction of Pyk2 siRNA in PC12 cells to investigate the function of Pyk2 in ionomycin-induced neurotransmitter release. Fig. 2A shows that PC12 cells transfected with 50 nM Pyk2 siRNA for 48 h caused a 90% decrease in Pyk2 protein abundance compared to those with negative control transfection. According to a previous study, Pyk2 expression is elevated in fibroblasts cultured from FAK^−/−^ mice [34]. Thus, FAK expression was tested in Pyk2 siRNA-treated cells. However, the compensatory response of FAK was not observed in this experiment. We transfected PC12 cells with 50 nM Pyk2 siRNA for 24, 48, and 72 h, and the result indicated that Pyk2 expression was inhibited by Pyk2 siRNA in a time-dependent manner. When PC12 cells were transfected with 10, 50, and 100 nM Pyk2 siRNA for 48 h, Pyk2 expression was inhibited in a dose-dependent manner. Thus, Pyk2 was specifically inhibited by Pyk2 siRNA in this study. The effect of Pyk2 siRNA on ionomycin-induced DA release was also investigated. PC12 cells were transfected with 100 nM Pyk2 siRNA, maintained for 48 h, and stimulated by 1 μΜ ionomycin. Fig. 2B shows that Pyk2 siRNA significantly abolished DA release compared with negative control siRNA (0 min to 2 min groups, one way ANOVA, *post-hoc* Newman-Keuls, F_3,28_ = 346.2, ***p<0.001 Pyk2 siRNA+iono *vs.* Control siRNA+iono). The result confirmed the involvement of Pyk2 in ionomycin-induced neurotransmitter release.

### Pyk2 Tyrosine Phosphorylation is Site-specific only for Tyr-402, and is Immediately Induced by Ionomycin

**Figure 3:**
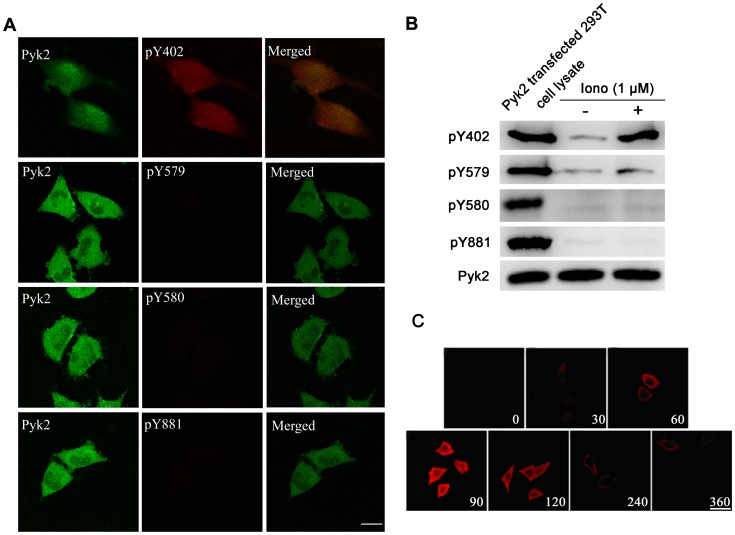
Ionomycin-induced Pyk2 tyrosine phosphorylation is site-specific only for Tyr-402. (**A**) PC12 cells were exposed to 1 μM ionomycin at 37°C for 90 s. The cells were fixed, and phosphotyrosine proteins were labeled by phosphorylation site-specific antibodies against Tyr-402 (pY402), Tyr-579 (pY579), Tyr-580 (pY580), or Tyr-881 (pY881) and TRITC-conjugated second antibody (red), respectively. Pyk2 expression was labeled by FITC (green). Scale bar, 5 μm. (**B**) PC12 cells were treated with or without 1 μM ionomycin at 37°C for 90 s, then lysed, and subjected to western blot using phosphorylation site-specific antibodies. Pyk2 transfected 293T cell lysate was used as a positive control. The total amount of Pyk2 was used as an internal control. (**C**) The time course of Pyk2 phosphorylation at Tyr-402 by ionomycin in PC12 cells. PC12 cells were exposed to 1 μM ionomycin at 37°C for the indicated times. The cells were labeled with pY402 site-specific polyclonal antibody, followed by a secondary layer of TRITC-conjugated second antibody to rabbit IgG (red). Scale bar, 10 μm.

Tyr-402, the major site of Pyk2 autophosphorylation upon activation, is essential for the induction of Pyk2 kinase activity, whereas Tyr-579, Tyr-580, and Tyr-881 further enhance Pyk2 kinase activity [18]. Due to the pronounced characteristics of Pyk2 tyrosine sites, we next addressed the effect of the phosphorylation state of these four tyrosine sites (Tyr-402, 579, 580, and 881) on ionomycin-mediated neurotransmission using double-immunolabeling and laser-scanning confocal microscopy (Fig. 3A). Tyr-402 phosphorylation was rapidly and markedly induced after treatment with 1 μΜ ionomycin in PC12 cells. By contrast, ionomycin-induced tyrosine phosphorylation for the other three Pyk2 tyrosine sites (Tyr-579, Tyr-580, and Tyr-881) was unaffected. We also confirmed this result through western blot using the specific anti-Pyk2 tyrosine site antibodies. Pyk2 transfected 293T cell lysate was used as a positive control (Fig. 3B). A further study illustrated that tyrosine phosphorylation on Tyr-402 after rapid intracellular calcium change was biphasic with an initial peak increase and decrease to baseline. Maximal Tyr-402 phosphorylation was achieved after 90 s (Fig. 3C), which was consistent with the results of Fig. 1E.

### Pyk2 Tyr-402 Autophosphorylation is Essential in Ionomycin-induced Neurotransmitter Release

**Figure 4:**
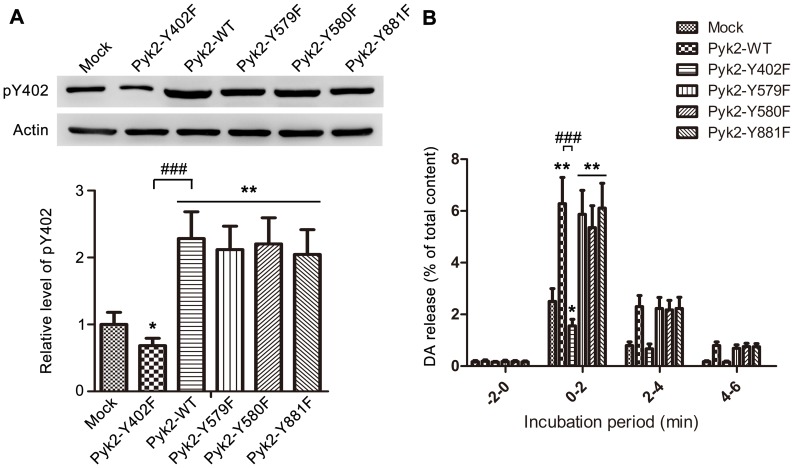
Pyk2 Tyr-402 autophosphorylation is essential in ionomycin-induced neurotransmitter release. (**A**) Phosphorylation of Pyk2 Tyr-402 in PC12 cells transfected with Pyk2-related plasmids. PC12 cells were transfected with 4 μg of empty vectors (Mock), Pyk2-WT, Pyk2-Y402F, Pyk2-Y579F, Pyk2-Y580F, and Pyk2-Y881F for 48 h. The cells were treated with 1 μM ionomycin for 2 min, and immediately harvested for analysis of Pyk2 Tyr-402 phosphorylation by western blot. Actin was used as an internal control. The average Pyk2 phosphorylation in the Mock group was set as 100% for all experiments to standardize the results with the same plasmids from repeated experiments (n = 4/sample). (**B**) PC12 cells were transfected with 4 μg of empty vector (Mock), Pyk2-WT, Pyk2-Y402F, Pyk2-Y579F, Pyk2-Y580F, and Pyk2-Y881F for 48 h, and used for DA release assay with 1 μM ionomycin. The amount of DA released in the medium was expressed as the percentage of the total cellular content (n = 8/sample). The values are expressed as means ± S.E.M. from four representative experiments (*p<0.05, **p<0.01,^ ###^p<0.001).

A series of Pyk2 mutants (Pyk2-Y402F, Pyk2-Y579F, Pyk2-Y580F, and Pyk2-Y881F) and the vector expressing wild-type Pyk2 (Pyk2-WT) were constructed to further investigate the role of Y402 in neurotransmitter release. The Pyk2 mutants and Pyk2-WT were individually transfected into PC12 cells. The transfection efficiencies exceeded 80% in PC12 cells (Fig. S2). Under ionomycin stimulation, Pyk2-Y402F transfection significantly inhibited the phosphorylation of Pyk2 Y402 compared with cells transfected with pcDNA3.1 empty vector (Mock) (Fig. 4A, one way ANOVA, *post-hoc* Newman-Keuls, F_5,18_ =  = 8.017, *p<0.05). Transfection of Pyk2-WT and other mutants (Pyk2-Y579F, Pyk2-Y580F, and Pyk2-Y881F) could induce significant phosphorylation on Y402 compared with the Mock group (**p<0.01). These results indicate that Y579, Y580, and Y881 did not affect Y402 phosphorylation during the short duration of ionomycin stimulation (Fig. 4A).

To investigate the function of different tyrosine sites of Pyk2 in ionomycin-induced DA release, the aforementioned vectors were transfected into PC12 cells. Fig. 4B shows that DA release was markedly enhanced by Pyk2-WT transfection, compared with DA release in mock group (0 min to 2 min, one way ANOVA, *post-hoc* Newman-Keuls, F_5,42_ = 43.02, **p<0.01), whereas Pyk2-Y402F transfection inhibited DA release compared with the mock (*p<0.05) and Pyk2-WT groups (^###^ *p*<0.001). Other mutants performed similarly to Pyk2-WT, with no statistically significant difference. These results were consistent with the phosphorylation level of Y402.

### PP2-enhanced Neurotransmitter Release is Pyk2-Tyr-402-dependent

**Figure 5:**
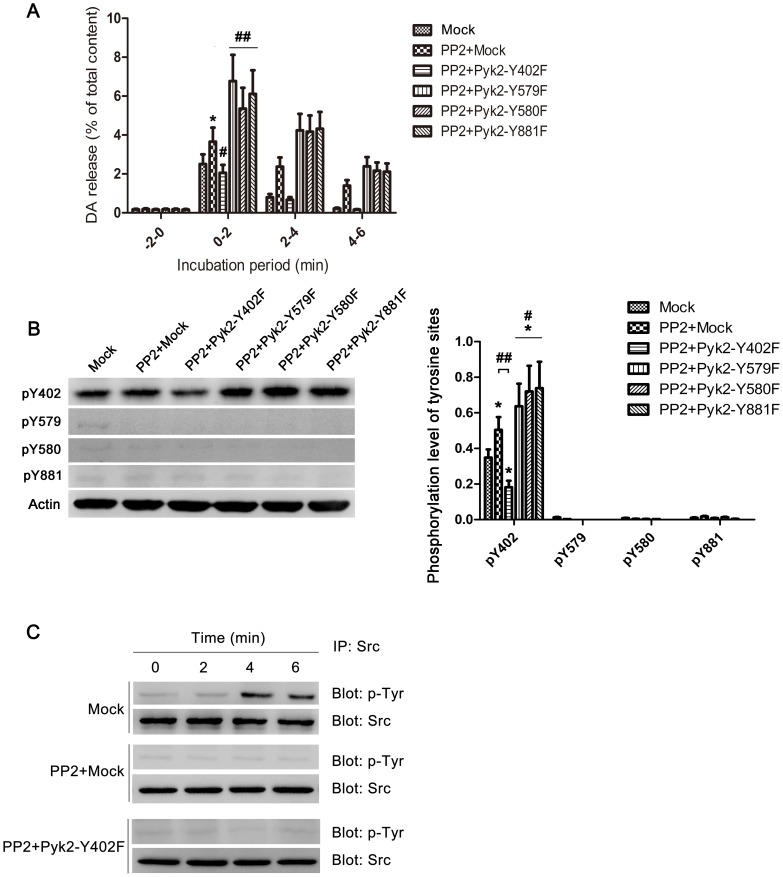
PP2-enhanced neurotransmitter release is Pyk2 Tyr-402-dependent. (**A**) PC12 cells were transfected with 4 μg of empty vectors (Mock), Pyk2-Y402F, Pyk2-Y579F, Pyk2-Y580F, and Pyk2-Y881F for 48 h, respectively. The cells were washed thrice with low-K^+^ solution, and incubated for 20 min in low-K^+^ solution with or without (Mock) 20 μM PP2 for 20 min. The cells were washed thrice, and sequentially incubated for 2 min in low-K^+^ solution with 1 μM ionomycin. The amount of DA release in the medium was expressed as the percentage of the total cellular content (n = 8/sample). (**B**) Another parallel performed groups after stimulated by ionomycin for 2 min were immediately harvested, and lysed for western blot using phosphorylation site-specific antibodies against Tyr-402 (pY402), Tyr-579 (pY579), Tyr-580 (pY580), or Tyr-881 (pY881). Actin was used as an internal control (n = 4/sample). (**C**) Equal amount of cell lysates harvested at different incubation period were immunoprecipitated with anti-Src antibody, then immunoblotted with anti-phosphotyrosine antibody. The total amount of Src was used as an internal control. The values are expressed as means ± S.E.M. from four representative experiments (*p<0.05, ^#^p<0.05, ^##^p<0.01, ^###^p<0.001).

Pyk2 autophosphorylation causes Src recruitment, whereas PP2, a src family tyrosine kinase inhibitor, can enhance Ca^2+^-dependent neurotransmitter release from neuronal cells [35]. We have also confirmed this report through contrasting the function of PP2 with PP3, an inactive PP2 analogue, in ionomycin evoked DA release, and found that PP2 enhanced DA release significantly compared with PP3, which had similar release level with control group (Fig. S3, one way ANOVA, *post-hoc* Newman-Keuls, F_2_,_21_ = 22.12, *P<0.05 PP2 *vs.* Control, ^#^ *p*<0.05 PP2 *vs.* PP3). PC12 cells were transfected with empty vector (Mock) and Pyk2 mutants to determine the function of different tyrosine sites of Pyk2 in PP2-enhanced neurotransmitter release. Fig. 5A shows that PP2 addition in PC12 cells could enhance DA release compared with that in the mock group (0 min to 2 min, one way ANOVA, *post-hoc* Newman-Keuls, F_5,42_ = 25.94, *p<0.05 PP2+Mock *vs*. Mock), whereas the enhancement was removed when PC12 cells were transfected with Pyk2-Y402F (^#^ *p*<0.05 PP2+Pyk2-Y402F *vs*. PP2+Mock). By contrast, other mutants could further enhance PP2-induced DA release (^##^ *p*<0.01 *vs.* PP2+Mock). This result indicates that Y402 of Pyk2 was essential for PP2-induced DA release, and other tyrosine sites were not equally important as Y402 in this process. The phosphorylation state of Pyk2 at various tyrosine sites was monitored after 2 min of stimulation with ionomycin. The result confirmed that the phosphorylation changes in Y402 between different groups were the associated factors in this process (Fig. 5B, pY402, one way ANOVA, *post-hoc* Newman-Keuls, F_5,18_ = 18.12, *p<0.05 *vs*. Mock, ^#^ *p*<0.05 *vs*. PP2+Mock, ^##^ *p*<0.01 *vs*. PP2+Mock). The phosphorylation state of Src was also monitored. Src tyrosine kinase was activated at 4 min, and its activity was maintained until 6 min after ionomycin stimulation in the mock group; the added PP2 masked this tyrosine phosphorylation changes (Fig. 5C). Associated tyrosine phosphorylation changes of Src with the result in Fig. 5A, which indicated that PP2 addition group (except for PP2+Pyk2−Y402F group) still had higher DA release in 4 min to 6 min compared with the mock group (Mock), we want to know whether Src inhibition is related to Pyk2 Y402-involved release.

### Src siRNA Prolongs Pyk2 Autophosphorylation and Increases Neurotransmitter Release

**Figure 6:**
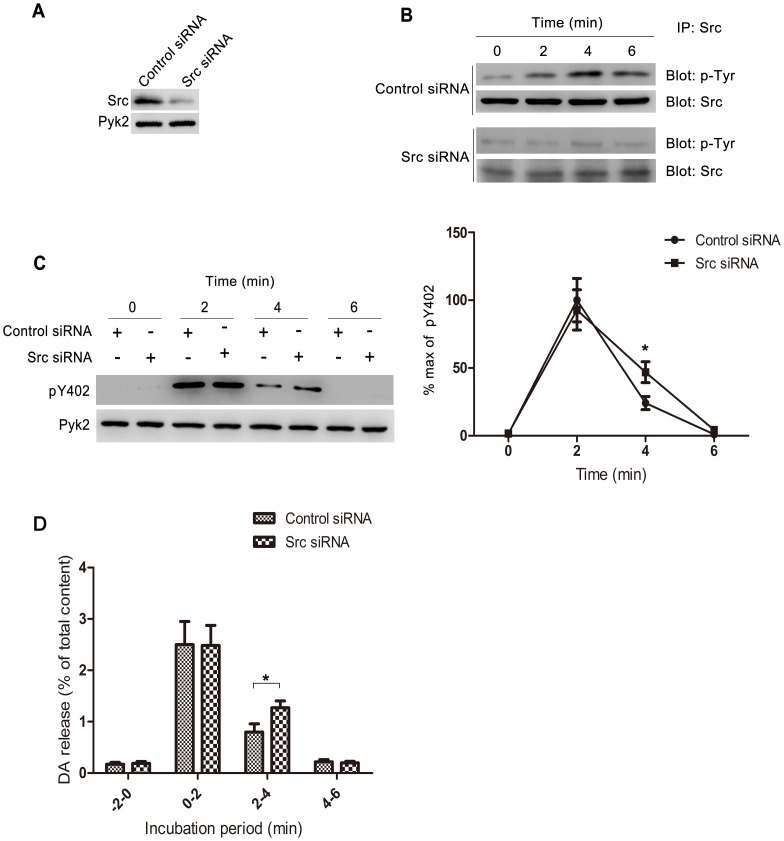
Src siRNA prolongs Pyk2 autophosphorylation and increases DA release. (**A**) PC12 cells were transfected with 100 nM Src siRNA or control siRNA for 48 h, then harvested, and lysed for western blot using anti-Src antibody and anti-Pyk2 antibody. (**B**) PC12 cells were transfected with 100 nM Src siRNA or control siRNA for 48 h, and incubated with 1 μM ionomycin for 2 min. Cells were harvested at 0, 2, 4, and 6 min after ionomycin treatment, then immunoprecipitated with anti-Src antibody, then immunoblotted with anti-phosphotyrosine antibody. The total amount of Src was used as an internal control. (**C**) PC12 cells were transfected with 100 nM Src siRNA or control siRNA for 48 h, and incubated with 1 μM ionomycin for 2 min. Cells were harvested at 0, 2, 4, and 6 min after ionomycin treatment for western blot to test the expression of pY402 and total Pyk2. Pyk2 Y402 phosphorylation at each indicated time is expressed as a percentage of the maximal level of induced phosphorylation. (**D**) PC12 cells were transfected with 100 nM Src siRNA or control siRNA for 48 h, and incubated with 1 μM ionomycin for 2 min. The amount of DA released in the medium was expressed as the percentage of the total cellular content. The values are expressed as mean ± S.E.M. from four representative experiments (*p<0.05).

To illustrate the relationship between Src and Pyk2 in ionomycin-induced neurotransmitter release, we used siRNA to inhibit Src expression. Fig. 6A shows that 100 nM Src siRNA could inhibit 90% of Src expression in PC12 cells, but did not affect Pyk2 expression. PC12 cells were stimulated with 1 μΜ ionomycin for 2 min after transfection with 100 nM Src siRNA or negative control siRNA for 48 h. The tyrosine phosphorylation of Src in different incubation periods was monitored by western blot. The result of the control siRNA is consistent with the previous observation in the Mock group (Fig. 5C). Src siRNA inhibited Src expression; thus, the modified tyrosine phosphorylation is shaded in Fig. 6B. Moreover, no statistical difference was observed in Pyk2 Y402 phosphorylation between the cells transfected with Src siRNA and negative control siRNA in 2 min. When the incubation time was extended to 4 min, the phosphorylation level of Pyk2 Y402 in Src siRNA-transfected cells was significantly higher than that in the negative control group (Fig. 6C, *p<0.05 Src siRNA *vs*. control siRNA in 2 min to 4 min, Student’s *t* test). Similarly, Src siRNA enhanced DA release in 2 min to 4 min (Fig. 6D, *p<0.05**Src siRNA *vs*. control siRNA in 2 min to 4 min, Student’s *t* test), which indicates that Src inhibition could extend the duration of Pyk2 autophosphorylation and increase neurotransmitter release.

## Discussion

Pyk2 can perform several post-synaptic functions, such as enhanced post-synaptic NMDA-R phosphorylation [24], [36], which contributes to LTP in CA1 hippocampus [24], and activation of the MAP kinase signal transduction pathway [36]. However, the functions of Pyk2 in pre-synaptic function, especially in neurotransmitter release, remain unclear.

Ca^2+^ triggers neurotransmitter release in at least two principal modes, synchronous and asynchronous release [31]–[33]. Synchronous release is triggered by brief localization Ca^2+^ waves induced by action potentials [37], whereas asynchronous release is triggered by increased bulk “residual” Ca^2+^ [38]–[40]. Ionomycin, a Ca^2+^ ionophore, when added to the medium will spontaneously incorporate into the neuronal plasma membrane, which allows Ca^2+^ to enter nerve terminals directly, and triggers asynchronous transmitter release. Our study revealed that Pyk2 (120 kDa, Figs. 1C and 1D) and paxillin (68 kDa, data not shown) were tyrosine-phosphorylated in PC12 cells after stimulation with ionomycin, which was consistent with previous reports in other cell lines [35]. Our results suggest that Pyk2 phosphorylation peaked at 1.5 min to 2 min, and returned to baseline after 5 min under Ca^2+^ stimulation in PC12 cells (Fig. 1E), which was concurrent with the DA release from PC12 cells (Fig. 1A) and Glu release from CGNs (Fig. 1B).

To determine whether Pyk2 is involved in ionomycin-induced neurotransmitter release, neurotransmitter release was observed when Pyk2 was inhibited by transfection of Pyk2 siRNA. Based on the high similarity and compensation between Pyk2 and FAK [34], we first tested their expression levels. The results show that 50 nM Pyk2 siRNA could inhibit 90% of Pyk2 expression, but had no effect on FAK expression, which suggests that Pyk2 siRNA was specific for Pyk2 (Fig. 2A). DA release was significantly inhibited in PC12 cells transfected with Pyk2 siRNA compared with that in cells transfected with negative control siRNA (Fig. 2B). To investigate the general effect of Pyk2 siRNA on PC12 cells, we detected the cell differentiation and general release machinery of PC12 cells treated with Pyk2 siRNA. Neither the control siRNA nor the Pyk2 siRNA induced remarkable cell differentiation, which is characterized by the absence of significant neurite outgrowth. PC12 cells tansfected with Pyk2 siRNA appeared rounder than those transfected with the control siRNA (Fig. S1). This phenomenon is consistent with the function of Pyk2, that is, organization of cytoskeletal components in a polarized manner for directional motility [41]. Besides, PC12 cells transfected with PKM (a kinase-inactive Pyk2 mutant) and PRNK (an alternatively spliced form of Pyk2 containing only the C-terminal tail of Pyk2) abolish differentiation for these two mutants inhibit the normal function of Pyk2 as the important sensor of EGF to induce PC12 cell differentiation [42]. Morever, as shown in Fig. 2B, the basal level of DA release (−2 min to 0 min) from PC12 cells did not change after Pyk2 siRNA transfection.

Pyk2 has four tyrosine sites (Y402, Y579, Y580, and Y881), among which Y402 was recognized as the main autophosphorylation site, and termed as the activated marker of Pyk2. To illustrate which site is involved in neurotransmitter release, site-specific phosphorylation antibody was used to test their phosphorylation degree individually. Our results demonstrate that ionomycin-induced calcium influx activated tyrosine phosphorylation of Pyk2 on Tyr-402 in PC12 cells, whereas tyrosine phosphorylation of the other three sites (Tyr-579, Tyr-580, and Tyr-881) did not cause any changes, at least during the monitor time (Fig. 3A and 3B). In addition, the kinetics of Y402 phosphorylation was also concurrent with neurotransmitter release (Figs. 1A and 1B), as shown by its maximum phosphorylation at 1.5 min and recovery after 5 min (Figs. 1E and 3C).

We constructed different vectors carrying Pyk2-WT and the four Pyk2 mutants (Pyk2-Y402F, Pyk2-Y579F, Pyk2-Y580F, and Pyk2-Y881F) to verify the function of different tyrosine sites on ionomycin-induced neurotransmitter release. As shown in Figs. 4B, overexpression of Pyk2-Y402F significantly decreased the DA release from PC12 cells. Besides, this finding also indicated that overexpression of exogenous Pyk2-Y402F affected the expression of endogenous Pyk2; this result is also found in previous study [43]. Three reasons may have contributed to this result. First, the viral promoters of eukaryotic expression vectors are generally more efficient than the promoters of a nuclear genome. This phenomenon can also be supported by normal results that the transcription levels are generally more efficient in a transient transfection system than in a stable transfection system. Second, overexpression of exogenous Pyk2 affects expression of endogenous Pyk2 through negative feedback regulation. Third, Pyk2-Y402F may form dimer with endogenous Pyk2, which further inhibits Y402 activation.

Based on our results, Pyk2-Y402 is essential in ionomycin-induced neurotransmitter release. However, Ohnishi [35] et al. found that PP2, an inhibitor of src family kinase, contributed to the neurotransmitter release. Pyk2 showed a decrease in tyrosine phosphorylation in PP2-treated cells, which was in conflict with our findings. We believe that this difference in results reflects the difference in our experimental procedures. For example, we used 1 μM ionomycin for 2 min to simulate calcium overload and the asynchronous neurotransmitter release, whereas Ohnishi et al. used 1 μM ionomycin for 5 min then tested the Pyk2 phosphorylation level. In our previous results, Pyk2 phosphorylation almost decreased to baseline at 5 min (Fig. 1C).

To illustrate the relationship between Pyk2 and PP2 in neurotransmitter release, we added PP2 to the PC12 cells transfected with four kinds of mutants. The result shows that Y402 mutant could abolish the effect of PP2 compared with other mutants. Thus, PP2 could enhance neurotransmitter release, but that was Pyk2-Y402-dependent (Fig. 5A).

Pyk2, an activator of Src, promotes neurotransmitter release, while PP2, an inhibitor of src family, also enhances neurotransmitter release. So what’s the role of Src in ionomycin-induced neurotransmitter release? According to our results, phosphorylation of Src occurred from 4 min to 6 min. Src inhibition (through PP2 or Src siRNA, Fig. 5C and 6B) could extend the duration of Pyk2 Y402 phosphorylation (Fig. 6C) and DA release from 2 min to 4 min (Fig. 5A and 6D). Besides, Ohnishi reported that PP2 enhanced Ca^2+^-dependent neurotransmitter release, and this process associated with dephosphorylation of Src and its substrate Pyk2 and paxillin [35]. Based on section five and six in our study, an interesting hypothesis is described as follows. Pyk2 is autophosphorylated by dimerization, which contributes to the phosphorylation of Pyk2 Y402, when intracellular Ca^2+^ concentrations are increased. Activated Pyk2 then facilitates neurotransmitter release by interacting with or activating synaptic-related proteins. Later, the enhancement of intracellular Ca^2+^ concentrations contribute to the activation of Src. Src further activates other tyrosine sites of Pyk2 and paxillin, which possibly contributes to the polymerization of actin skeleton [35], subsequently inhibits neurotransmitter release. Thus, Src and its substrates exhibit a negative feedback regulation of Ca^2+^-induced neurotransmitter release. An inhibitor of src family kinases, PP2 may contribute to neurotransmitter release by blocking this negative feedback (Fig. S5 shows a simple schematic diagram of this hypothesis). This hypothesis is consistent with Fig. 5. The activation of Pyk2 Y402 is a pre-requisite for PP2 to function in ionomycin-induced neurotransmitter release. Moreover, the inhibition of Src by Src siRNA prolongs the DA release at least to 4 min could also be explained. However, this hypothesis also presents several limitations. For example, other src family tyrosine kinases may participate in the negative feedback because PP2 could both significantly enhance and prolong the release compared with Src siRNA (Fig. 5A). Considering that PP2 is not the specific inhibitor of Src, we speculate that other factors may be involved after Pyk2 Y402 is activated. We also monitored the state of tyrosine phosphorylation of Fyn, which is another important src family tyrosine kinase abundantly expressed in the CNS. However, no relationship with this process was likely observed (Fig. S4). Further studies should be conducted to investigate this hypothesis.

This study showed that Pyk2 was involved in ionomycin-induced neurotransmitter release, and Y402 was specifically activated in this process. The function of PP2 in neurotransmitter release was Pyk2-Y402-dependent. Src inhibition could prolong the duration of neurotransmitter release. Pyk2 activation has been reported in pathological conditions of neurotoxicity, cerebral ischemia, and seizures [29], [44], [45]. Thus, our research proposes the possibility that Pyk2 overexpression may contribute to neurotransmitter disorders during stroke or neurotoxic conditions in Ca^2+^ overload. Further studies on Pyk2-induced neurotransmitter release may guide prospective therapeutic approaches for neurological disorders.

## Materials and Methods

### Ethics Statement

The experiments were approved by the Research Ethics Committee of the Chinese Academy of Medical Sciences and Peking Union Medical College (approval number: PUM201033334A). Animals were maintained and experiments were conducted in accordance with the Institutional Animal Care and Use Committee, Chinese Academy of Medical Sciences and Peking Union Medical College, and the 1996 Guide for the Care and Use of Laboratory Animals (Institute of Laboratory Animal Resources on Life Sciences, National Research Council, National Academy of Sciences, Washington DC).

### Antibodies and Reagents

The following antibodies were used: rabbit polyclonal Pyk2 phosphorylation site-specific antibody (pY402, pY579, pY580, and pY881) from Biosource International (Camarillo, CA, USA); anti-phosphotyrosine monoclonal antibody (clone 4G10) from Upstate Biotechnology (Lake Placid, NY, USA); mouse monoclonal anti-Pyk2, actin antibodies, rabbit polyclonal anti-FAK, Src, actin, Fyn antibodies, horseradish peroxidase (HRP)-conjugated anti-mouse IgG, anti-rabbit IgG, rhodamine (TRITC)-conjugated anti-rabbit IgG, FITC-conjugated anti-mouse IgG, and Human Pyk2 transfected 293T whole cell lysate (sc-115595) were obtained from Santa Cruz Biotechnology (Santa Cruz, CA, USA).

Dulbecco’s modified eagle medium (DMEM), and reduced serum medium Opti-MEM were purchased from Gibco (Gibco, Carlsbad, CA, USA). Lipofectamine LTX and Lipofectamine RNAiMAX were purchased from Invitrogen (Invitrogen, Carlsbad, CA, USA). Fetal bovine serum (FBS) and horse serum were obtained from Hyclone (Rockford, IL, USA). Ionomycin was obtained from Sigma (Sigma, St. Louis, MO, USA). Mouse Nerve Growth Factor for Injection (NGF) was obtained from Staidson (Staidson, Beijing, China). Polyvinylidene fluoride (PVDF) membrane was obtained from Millipore (Bedford, MA), and protein G-Sepharose was obtained from Amersham Pharmacia Biotech. Prolong antifade kit was purchased from Molecular Probes (Carlsbad, CA, USA). Other materials were obtained from standard suppliers or as indicated in the text.

### Vector Construction and Small Interfering RNA (siRNA)

Pyk2 cDNA was amplified from RNA of rat pheochromocytoma (PC12) cells by PCR using the following composite primers encoding both ends of the rat Pyk2 open reading frame flanked with EcoRI and XhoI adapter as follows: 5′-CCGGAATTCGCCACCATGTCCGGGGTGTCTGA-3′ (sense) and 5′-CCGCTCGAGTCACTCTGCAGGCGGGT-3′ (antisense).

The Pyk2 cDNA fragment was cloned into pMD18T vector, and then subcloned into the pcDNA3.1 vector using EcoRI and XhoI.

The mutants of Pyk2 (Pyk2-Y402F, Y579F, Y580F, and Y881F) were generated by replacing tyrosine 402, 579, 580, and 881 with phenylalanine using site-directed mutagenesis, according to the manufacturer′s protocol (TransGen Biotech, Easy Mutagenesis System, FM101). Primers designed for these mutants were as follows:

Pyk2-Y402F: 5′-AGCATAGAGTCAGACATCT**T**TGCAGAGATT-3′ (sense),

5′-**A**AGATGTCTGACTCTATGCTACAGCTTTCT-3′ (antisense);

Pyk2-Y579F: 5′-ACATTGAGGATGAGGACT**T**TTACAAAGCTT-3′ (sense),

5′-**A**AGTCCTCATCCTCAATGTACCGGGAGA-3′ (antisense);

Pyk2-Y580F: 5′-ATTGAGGATGAGGACTATT**T**CAAAGCTTCCG-3′ (sense),

5′-**A**AATAGTCCTCATCCTCAATGTACCGGGAGA-3′ (antisense);

Pyk2-Y881F: 5′-ACAGGACTGATGACCTCGTGT**T**CCACAATGTCA-3′ (sense),

5′-**A**ACACGAGGTCATCAGTCCTGTCCAAGTT-3′ (antisense).

The mutations were confirmed by sequence analysis.

Pyk2 siRNA, Src siRNA, and their control siRNA were purchased from Santa Cruz Biotechnology, Inc. siRNA products generally consist of pools of three to five target-specific 19 nt to 25 nt siRNAs designed for knockdown of gene expression.

### Cell Culture

PC12 cells were plated on polyethylenimine-coated four-well plates (Nalge Nunc International, Rochester, NY, USA) at a density of 5×10^5^ cells/cm^2^, and maintained in DMEM with 5% heat-inactivated FBS and 5% horse serum at 37°C in a humidified atmosphere with 10% CO_2_.

Cerebellar granule cells were dissected from seven-day-old Wistar rats (Vital River Laboratory Animal Technology, Beijing, China), and incubated in 0.2% trypsin and 0.02% DNase I for 15 min at 37°C. The trypsinized tissue was triturated with a Pasteur pipette until no tissue aggregates were observed. The cells were then washed with DMEM containing 10% heat-inactivated FBS, and plated on polyethylenimine-coated four-well plates at a density of 5×10^5^ cells/cm^2^. The cells were maintained in culture medium [DMEM supplemented with 26 mM KCl, 1 mM cytosine arabinonucleoside (Sigma), 50 U/ml penicillin (Sigma), and 100 μg/ml streptomycin (Sigma)] containing 10% heat-inactivated FBS at 37°C in a humidified 10% CO_2_ atmosphere. Experiments were conducted after 19 d to 20 d of culture [35].

### Cell Differentiation

PC12 cells were washed twice with PBS and resuspended in serum-free DMEM for 1 h at a concentration of 2×10^5^ cells/cm^2^. Thereafter cells received NGF (100 ng/ml), and were immediately plated on poly-L-lysine coated dishes. Cells undergoing differentiation were observed after 2 days [42].

### Transfection

Two days before the experiments, cells were plated on poly-L-lysine-coated four-well culture dishes at a density of 1.0×10^6^ cells/well, and cultured for 24 h before transfection of plasmid with Lipofectamine or RNA oligonucleotides with Lipofectamine RNAiMAX according to the manual (Invitrogen, Carlsbad, CA, USA). The medium was changed after 6 h. Cells were used for the experiments after 48 h unless otherwise indicated in the text.

Co-transfection was performed to investigate the transfection efficiency. According to the procedure above, PC12 cells were transfected with 4 μg of pcDNA3.1 and 0.4 μg of pEGFP-N1, which expresses green fluorescence protein (GFP), in each well of six-well culture dishes. The green fluorescence was tested by immunofluorescence microscopy after 48 h. A total of 700 to 800 cells were counted in six to eight representative fields to calculate the transfection efficiency.

### Release Assay

The release assays were conducted at 37°C. Cells were washed thrice with low-K^+^ solution (140 mM NaCl, 4.7 mM KCl, 1.2 mM KH_2_PO_4_, 2.5 mM CaCl_2_, 1.2 mM MgSO_4_, 11 mM glucose, 15 mM Hepes-NaOH, pH 7.4). After pretreatment in various conditions as indicated in the figure legends, the cells were incubated for 2 min in low-K^+^ solution. The release of neurotransmitters during this period represented the basal release (−2 min to 0 min). The buffer was immediately changed after 2 min with low-K^+^ solution containing 1 μM ionomycin to stimulate Ca^2+^ influx (0 min to 2 min). The buffer was changed every 2 min with low-K^+^ solution. Sample buffer solutions were immediately collected into microtubes on ice at the end of each incubation period. For DA detection, the solutions were transferred to a microtube containing 100 μl of 0.1 M perchloric acid (PCA) on ice. After the experiments, the cells were sonicated on ice in 500 μl of chilled 40 mM PCA and 0.1 mM EDTA. The samples were centrifuged at 15,000 rpm for 5 min at 4°C, and the supernatant was stored at −80°C until use. The secreted amount of DA was assayed by high-performance liquid chromatography (HPLC) using a C_18_ column (3.2×150 mm, ESA, Inc., Chelmsford, MA, USA) and an electrochemical detector system (Model 5300 coulochem III, ESA, Inc.). For glutamate (Glu) measurement, samples were determined by reverse-phase HPLC on an Atlantis dC18 column (2.1×100 mm, Waters Inc., Milford, MA, USA) using pre-column derivatization with o-phthalaldehyde and fluorescence detection (G1321A, Agilent Technologies, Inc., Santa Clara CA, USA).

### Immunoprecipitation (IP) and Immunoblotting (IB) Analysis

Whole cells were lysed in ice-cold buffer [50 mM Tris·HCl, pH 7.5, 150 mM NaCl, 1% Nonidet P-40, 1 mM EDTA, 10 mM sodium pyrophosphate, 1% sodium deoxycholate, 0.1% sodium dodecyl sulfate (SDS), 10% glycerol] containing protease inhibitors (1 mM phenylmethylsulfonyl fluoride, 1 μg/ml pepstatin, 1 μg/ml leupeptin, and 10 μg/ml aprotinin) and phosphatase inhibitors (10 mM NaF and 1 mM Na_3_VO_4_). Lysates were clarified by centrifugation at 12,000 rpm for 30 min at 4°C, and the supernatants were incubated overnight at 4°C with the indicated antibodies, followed by protein G-Sepharose incubation for 2 h to 3 h at 4°C, and three washes with lysis buffer. Total cell lysates or immunoprecipitated proteins were separated on 8% SDS–polyacrylamide gel electrophoresis, and transferred onto PVDF membranes with a semi-dry transblotting apparatus. The membranes were blocked in 3% (w/v) bovine serum albumin in Tris-buffered saline containing 0.05% Tween-20, which was incubated subsequently with primary antibodies, followed by HRP-conjugated secondary antibodies, and developed with enhanced chemiluminescence and a luminescent image analyzer (LAS-3000; Fujifilm, Tokyo, Japan). For reprobing, selected membranes were stripped off antibodies by incubation in restore stripping buffer at room temperature for 10 min, followed by washing, blocking, and antibody incubation as described above.

### Immunofluorescence Microscopy

PC12 cells were prepared on pre-coated glass coverslips. After pretreatment in various conditions, the cells were fixed with 4% paraformaldehyde in PBS for 15 min at room temperature, permeabilized with 0.5% Triton X-100 in PBS for 10 min, and blocked with 3% goat serum in PBS containing 0.1% Tween-20 for 1 h at room temperature. The cells were then double-stained with a mouse monoclonal antibody and rabbit polyclonal antibody using FITC-goat anti-mouse IgG and TRITC-goat anti-rabbit IgG as secondary antibodies. Specimens were washed thrice with PBS, mounted with prolong antifade reagent, and observed with a confocal laser scanning microscope (TCS SP2, Leica, Solms, Germany).

### Statistical Analysis

All values are presented as means ± S.E.M. Each experiment was repeated at least thrice. Statistical analysis was performed by Student’s *t* test or one-way ANOVA analysis followed by pair-wise comparisons with Newman-Keuls test. A value of *p*<0.05 was considered significant. All statistical analyses were performed using SPSS13.0 software (Chicago, IL).

## Supporting Information

Figure S1 **Contrasting the morphology of treated PC12 cells. (A)** PC12 cells were transfected with 100 nM control siRNA for 48 h. **(B)** PC12 cells were transfected with 100 nM Pyk2 siRNA for 48 h. **(C)** PC12 cells were differentiated with 100 ng/ml NGF for 48 h. Scale bar, 10 μm.
(TIF)

Figure S2 **Transfection efficiency of pcDNA3.1 vector in PC12 cells.** Co-transfection was performed in PC12 cells with 4 μg of pcDNA3.1 and 0.4 μg of pEGFP-N1 in each well of six-well culture dishes. The green fluorescence was tested by immunofluorescence microscopy after 48 h. A total of 700 to 800 cells were counted in six to eight representative fields to calculate the transfection efficiency. Scale bar, 10 μm.
(TIF)

Figure S3 **Contrasting the effect of PP2 with PP3 on ionomycin-induced DA release.** PC12 cells were washed thrice with low-K^+^ solution, and incubated for 20 min in low-K^+^ solution with or without (Control) 20 μM PP2 or PP3 for 20 min. The cells were washed thrice, and sequentially incubated for 2 min in low-K^+^ solution with 1 μM ionomycin. Sample buffer solutions were immediately collected into microtubes on ice after 2 min of incubation period. The amount of DA release in the medium was expressed as the percentage of the total cellular content. The values are expressed as means ± S.E.M. from four representative experiments, n = 8/sample (*P<0.05 PP2 vs. Control,^ #^p<0.05 PP2 vs. PP3).
(TIF)

Figure S4 **Phosphorylation state of Fyn in section five.** PC12 cells were transfected with 4 μg of empty vectors (Mock, PP2+ Mock) or Pyk2-Y402F for 48 h. The cells were washed three times with low-K^+^ solution, and incubated for 20 min in low-K^+^ solution with or without (Mock) 20 μM PP2 for 20 min. The cells were washed thrice, and sequentially incubated for 2 min in low-K^+^ solution with 1 μM ionomycin. An equal amount of cell lysates harvested at different incubation periods was immunoprecipitated with anti-Fyn antibody, and immunoblotted with anti-phosphotyrosine antibody. The total amount of Fyn was used as an internal control.
(TIF)

Figure S5 **Schematic diagram of the hypothesis about Pyk2-involved neurotransmitter release.** Increased intracellular Ca^2+^ concentration causes Pyk2 Y402 autophosphorylation by dimerization. Activated Pyk2 facilitates neurotransmitter release potentially through interacting with or activating synaptic-related proteins. On the other side, increased intracellular Ca^2+^ concentration also activates Src, which further activates other tyrosine sites of Pyk2 and paxillin, and then contributes to the polymerization of actin skeleton. Neurotransmitter release was inhibited as the result. Thus, Src and its substrates form a negative feedback regulation of Ca^2+^ induced neurotransmitter release. PP2, the inhibitor of src family kinases, may contribute to neurotransmitter release though inhibiting this negative feedback.
(TIF)
