# Supplementary figures and images for: Tyrosine 402 Phosphorylation of Pyk2 Is Involved in Ionomycin-Induced Neurotransmitter Release
Source: PLoS One. 2014 Apr 9;9(4):e94574. doi: 10.1371/journal.pone.0094574 (PMC3981813; doi:10.1371/journal.pone.0094574)

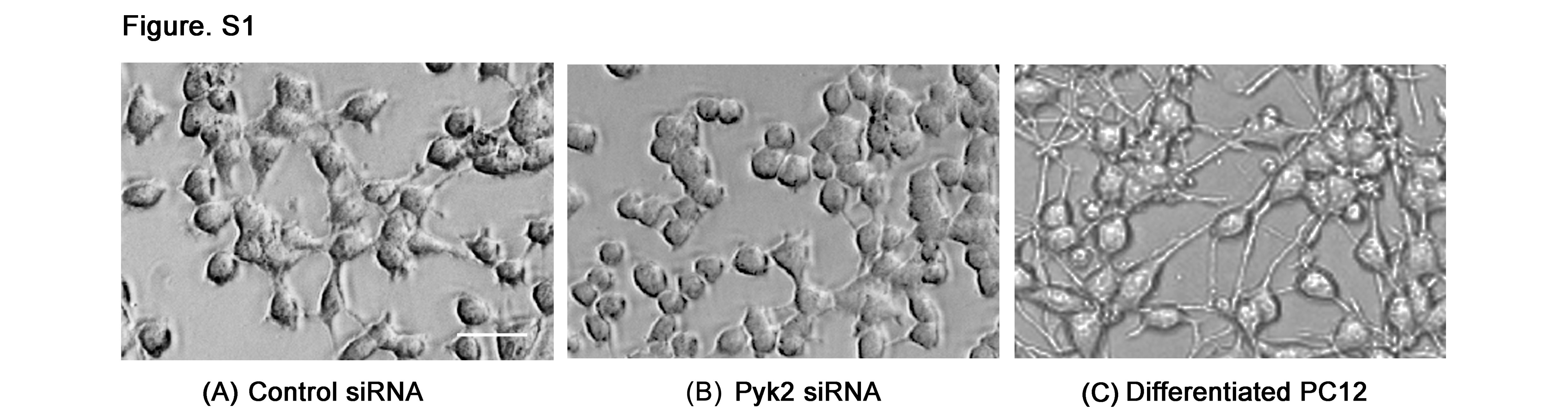

Supplement: Figure S1 — Contrasting the morphology of treated PC12 cells. (A) PC12 cells were transfected with 100 nM control siRNA for 48 h. (B) PC12 cells were transfected with 100 nM Pyk2 siRNA for 48 h. (C) PC12 cells were differentiated with 100 ng/ml NGF for 48 h. Scale bar, 10 μm. (TIF) [file pone.0094574.s001.tif]

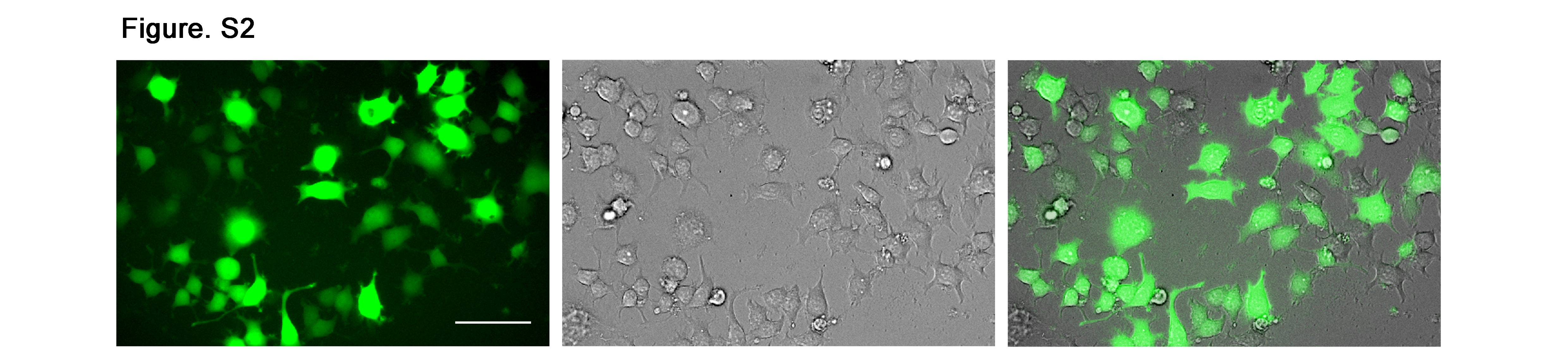

Supplement: Figure S2 — Transfection efficiency of pcDNA3.1 vector in PC12 cells. Co-transfection was performed in PC12 cells with 4 μg of pcDNA3.1 and 0.4 μg of pEGFP-N1 in each well of six-well culture dishes. The green fluorescence was tested by immunofluorescence microscopy after 48 h. A total of 700 to 800 cells were counted in six to eight representative fields to calculate the transfection efficiency. Scale bar, 10 μm. (TIF) [file pone.0094574.s002.tif]

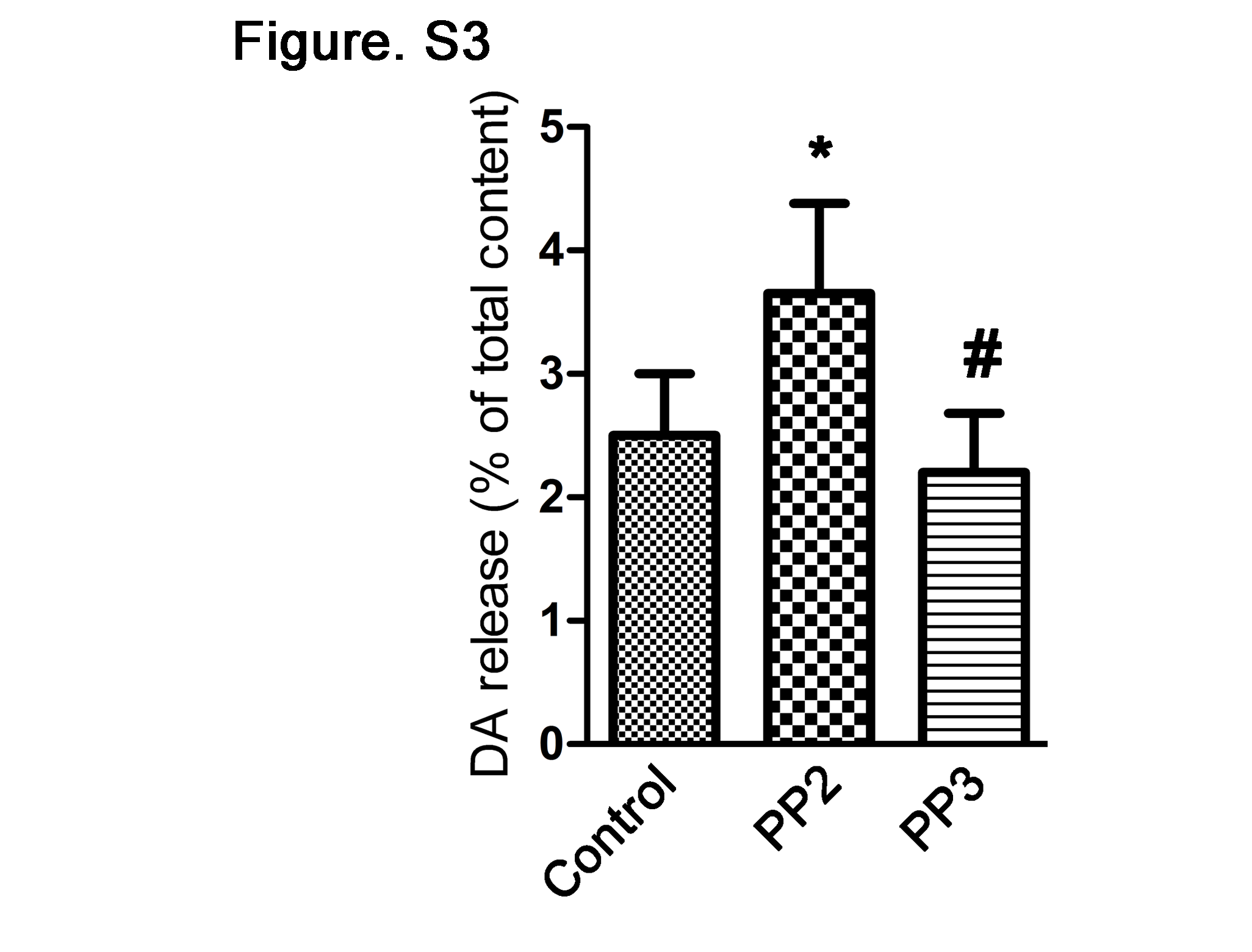

Supplement: Figure S3 — Contrasting the effect of PP2 with PP3 on ionomycin-induced DA release. PC12 cells were washed thrice with low-K+ solution, and incubated for 20 min in low-K+ solution with or without (Control) 20 μM PP2 or PP3 for 20 min. The cells were washed thrice, and sequentially incubated for 2 min in low-K+ solution with 1 μM ionomycin. Sample buffer solutions were immediately collected into microtubes on ice after 2 min of incubation period. The amount of DA release in the medium was expressed as the percentage of the total cellular content. The values are expressed as means ± S.E.M. from four representative experiments, n = 8/sample (*P<0.05 PP2 vs. Control, #p<0.05 PP2 vs. PP3). (TIF) [file pone.0094574.s003.tif]

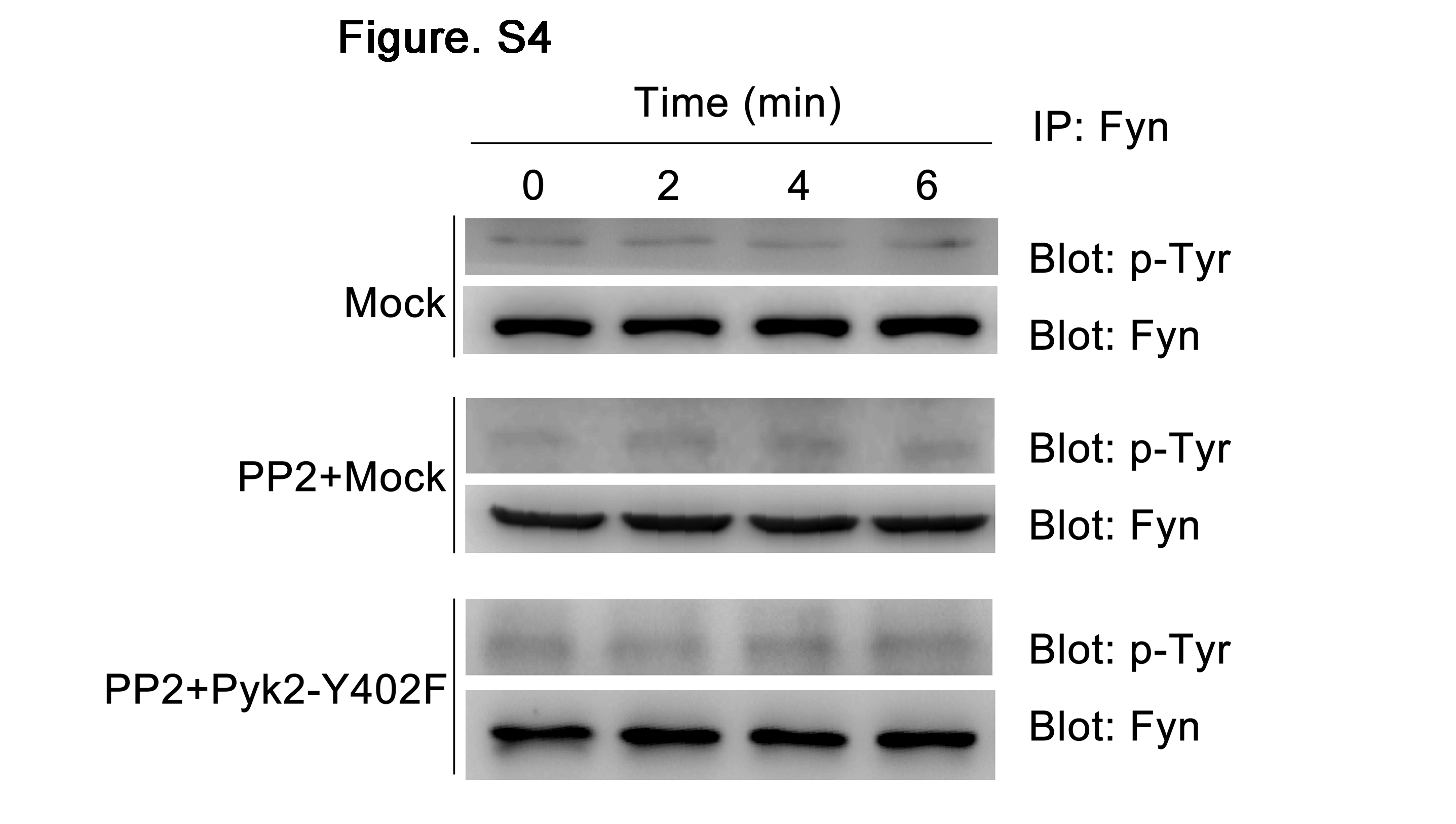

Supplement: Figure S4 — Phosphorylation state of Fyn in section five. PC12 cells were transfected with 4 μg of empty vectors (Mock, PP2+ Mock) or Pyk2-Y402F for 48 h. The cells were washed three times with low-K+ solution, and incubated for 20 min in low-K+ solution with or without (Mock) 20 μM PP2 for 20 min. The cells were washed thrice, and sequentially incubated for 2 min in low-K+ solution with 1 μM ionomycin. An equal amount of cell lysates harvested at different incubation periods was immunoprecipitated with anti-Fyn antibody, and immunoblotted with anti-phosphotyrosine antibody. The total amount of Fyn was used as an internal control. (TIF) [file pone.0094574.s004.tif]

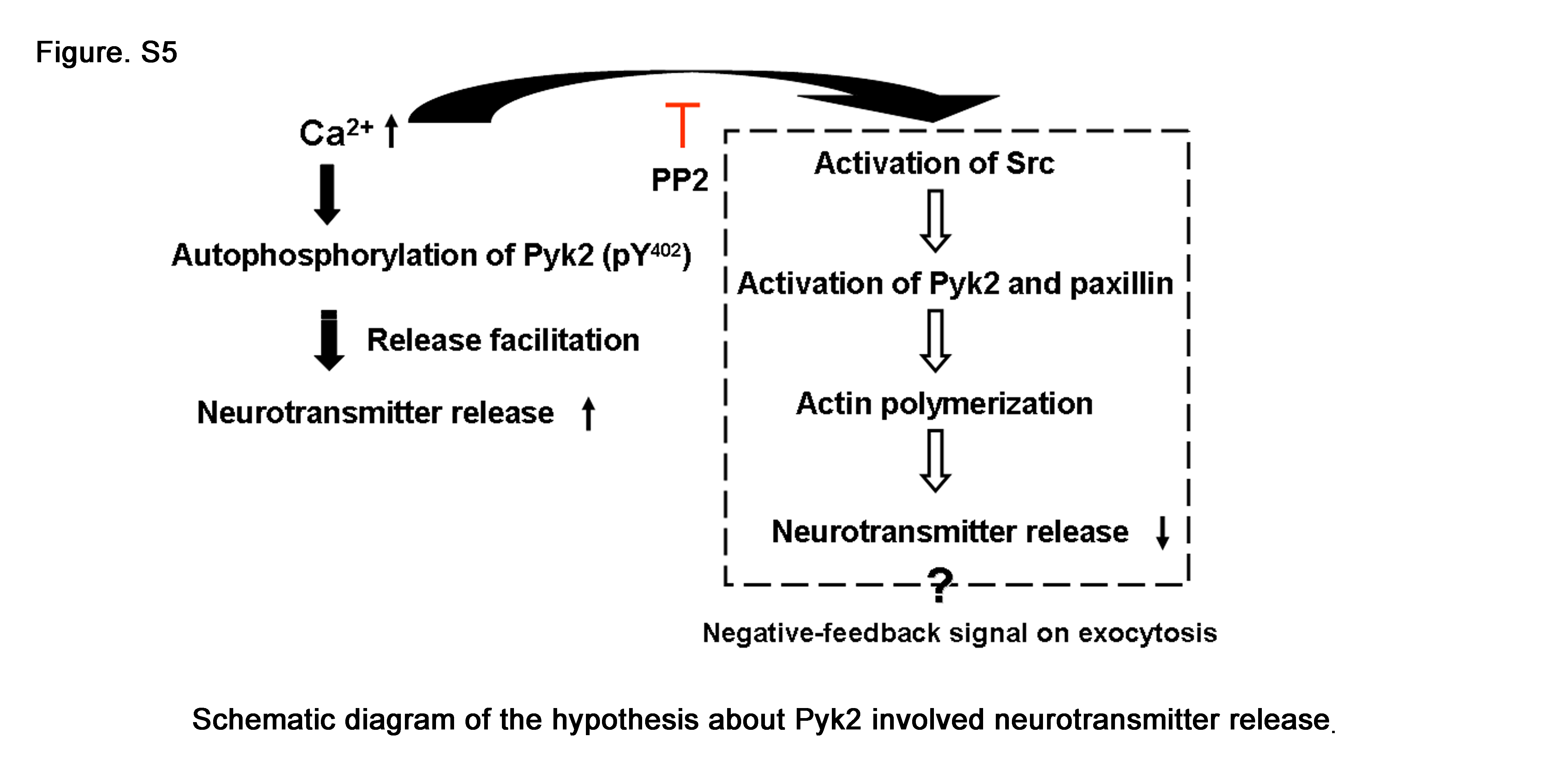

Supplement: Figure S5 — Schematic diagram of the hypothesis about Pyk2-involved neurotransmitter release. Increased intracellular Ca2+ concentration causes Pyk2 Y402 autophosphorylation by dimerization. Activated Pyk2 facilitates neurotransmitter release potentially through interacting with or activating synaptic-related proteins. On the other side, increased intracellular Ca2+ concentration also activates Src, which further activates other tyrosine sites of Pyk2 and paxillin, and then contributes to the polymerization of actin skeleton. Neurotransmitter release was inhibited as the result. Thus, Src and its substrates form a negative feedback regulation of Ca2+ induced neurotransmitter release. PP2, the inhibitor of src family kinases, may contribute to neurotransmitter release though inhibiting this negative feedback. (TIF) [file pone.0094574.s005.tif]
